# Comment on: Effect of psilocybin therapy on suicidal ideation, attempts, and deaths in people with psychiatric diagnoses: a systematic review and meta-analysis

**DOI:** 10.1177/20451253251397590

**Published:** 2025-12-03

**Authors:** Tiago Machado, Alexandra S. L. Rodrigues, João Costa

**Affiliations:** Laboratório de Farmacologia Clínica e Terapêutica, Faculdade de Medicina, Universidade de Lisboa, Avenida Prof. Egas Moniz, Lisbon 1649-028, Portugal; Neuroradiology Department, Unidade Local de Saúde São José, Lisbon, Portugal; Laboratório de Farmacologia Clínica e Terapêutica, Faculdade de Medicina, Universidade de Lisboa, Lisbon, Portugal

**Keywords:** meta-analysis, psilocybin, psychedelics, suicidal ideation, suicide, suicide attempts, systematic review

The systematic review by Wong et al.^1^ provides a timely and relevant evidence synthesis on suicidality outcomes associated with psilocybin therapy, given the uncertainty around this topic. The authors calculated pooled standardized mean differences (SMDs) and reported a significantly small effect in favor of psilocybin therapy in reducing suicidal ideation versus controls. Although SMD values presented in the text and figure differ slightly, the overall conclusion is that psilocybin may reduce suicidal ideation. We were, however, surprised to see almost all individual trials contributing effect sizes in the same direction, as the evidence from primary studies has not been consistent on this outcome. This prompted us to re-examine the data.

In Goodwin et al.,^2^ which carries the greatest weight in the meta-analysis, mean scores on the Columbia-Suicide Severity Rating Scale increased in all groups, showing a minor worsening in suicidal ideation. This worsening was numerically greater in the psilocybin groups, yet, in the forest plot by Wong et al. (p. 6), the trial is shown with a negative effect size, suggesting reduced suicidality for psilocybin compared with control. This discrepancy could substantially affect the direction of the pooled result. Similar inconsistencies are seen in other included studies. For Rosenblat et al.,^3^ the review lists mean values for the suicidal ideation item of the Montgomery-Åsberg depression rating scale that we could not locate in the cited publication. According to the authors, the control group showed a greater numerical reduction in ideation, but, again, the forest plot shows the opposite direction, favoring psilocybin. Likewise, for Ross et al.,^4,5^ the forest plot indicates an effect favoring control, in the opposite direction from what is described in subsequent reporting. The metric used to derive these SMDs, whether based on change-from-baseline or another approach, is not clearly specified. Together, these issues raise concerns about the validity of the review’s main finding.

We replicated the meta-analysis using the data provided in the supplementary material by Wong et al., complemented by values verified from the original publications whenever possible.^2–9^ A random-effects model (inverse-variance method) was used to derive SMD (change-from-baseline scores). Pooled SMD was −0.07 (95% CI: −0.25 to 0.10), indicating no significant difference in suicidal ideation between psilocybin and control (Figure 1). Our recalculation underscores the importance of transparent analysis and reporting.

**Figure 1.**
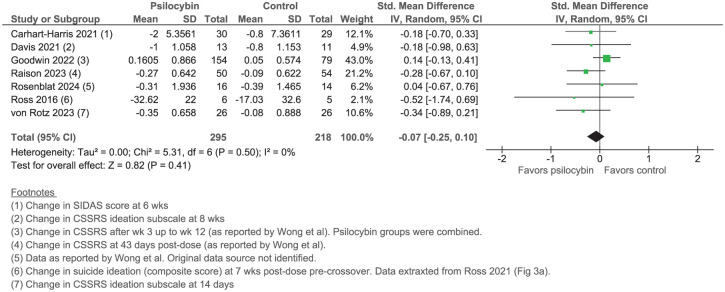
Replication of Wong et al. meta-analysis.

Individual trials point in different directions, reflecting the complexity of this question. In Goodwin et al.,^2^ a higher proportion of participants in psilocybin arms showed a worsening of suicidal state, and three aborted suicide attempts occurred in the 25 mg group, findings that could not be captured by this type of analysis. Such findings further underscore the need for caution in interpreting the evidence, as pooled analysis may fail to capture the nuances of suicidality outcomes. Moreover, psilocybin therapies are nearly impossible to blind effectively, and suicidality scales rely on subjective assessments, contributing to a high risk of bias and very low certainty in the evidence.^10^ Overall, the available evidence is insufficient to support any firm conclusions about the effect of psilocybin on suicidality.

## Declarations

**Ethics approval and consent to participate:** Not applicable.

**Consent for publication:** Not applicable.

**Availability of data and materials:** Not applicable.
